# Selection of Membrane RNA Aptamers to Amyloid Beta Peptide: Implications for Exosome-Based Antioxidant Strategies

**DOI:** 10.3390/ijms20020299

**Published:** 2019-01-13

**Authors:** Teresa Janas, Karolina Sapoń, Michael H. B. Stowell, Tadeusz Janas

**Affiliations:** 1 Institute of Biotechnology, University of Opole, Kominka 6, 45-032 Opole, Poland; teresa.janas@uni.opole.pl (T.J.); karolina.sapon@uni.opole.pl (K.S.); 2 Department of MCD Biology, University of Colorado, Boulder, CO 80309, USA; michael.stowell@colorado.edu; 3 Mechanical Engineering, University of Colorado, Boulder, CO 80309, USA

**Keywords:** Alzheimer’s disease, amyloid, Down’s syndrome, exosomes, liposomes, oxidative stress, phosphatidylserine, rafts, RNA aptamers, SELEX

## Abstract

The distribution of amyloid beta peptide 42 (Aβ42) between model exosomal membranes and a buffer solution was measured. The model membranes contained liquid-ordered regions or phosphatidylserine. Results demonstrated that up to ca. 20% of amyloid peptide, generated in the plasma (or intracellular) membrane as a result of proteolytic cleavage of amyloid precursor proteins by β- and γ-secretases, can stay within the membrane milieu. The selection of RNA aptamers that bind to Aβ42 incorporated into phosphatidylserine-containing liposomal membranes was performed using the selection-amplification (SELEX) method. After eight selection cycles, the pool of RNA aptamers was isolated and its binding to Aβ42-containing membranes was demonstrated using the gel filtration method. Since membranes can act as a catalytic surface for Aβ42 aggregation, these RNA aptamers may inhibit the formation of toxic amyloid aggregates that can permeabilize cellular membranes or disrupt membrane receptors. Strategies are proposed for using functional exosomes, loaded with RNA aptamers specific to membrane Aβ42, to reduce the oxidative stress in Alzheimer’s disease and Down’s syndrome.

## 1. Introduction

Oxidative stress occurs early in the course of Alzheimer’s disease (AD), which would support its key role in AD pathogenesis, in relation with the presence of amyloid peptide (Aβ) [1]. Elevated levels of Aβ42 have been reported to be associated with increased levels of oxidation products from proteins, lipids and nucleic acids in AD hippocampus and cortex. In contrast, brain regions with low Aβ levels (e.g., cerebellum) did not exhibit high concentrations of oxidative stress markers. It has been confirmed that membrane proteins were more oxidatively damaged than cytoplasmic proteins in the human brain [1].

The toxicity of amyloid peptides is correlated with their interactions with cell membranes [2], and with the production of reactive oxygen species (ROS) near the plasma membrane due to the accumulation of Cu, Zn and Fe ions in the amyloid plaques [1]. Amyloid peptides can enter the membrane from the external solution, and membrane surfaces promote the conversion of amyloid peptides into toxic aggregates. The ganglioside content, cholesterol content, and overall fluidity (ordered raft regions versus disordered non-raft regions) of membranes have all been shown to be important factors in the anchoring of amyloidogenic proteins to membranes [2]. On the other hand, it is possible that a fraction of these peptides never leave the membrane lipid bilayer after they are generated, but instead exert their toxic effects by competing with and compromising the functions of intramembranous segments of membrane-bound proteins [3]. Evidence indicates that the toxicity of amyloid peptides is directly correlated with their ability to form ion channels or pores that cause membrane depolarization, Ca^2+^ leakage, and a disruption of ionic homeostasis. It is also possible that a fraction of amyloid peptides do not fully penetrate the membrane, but rather associate with membrane surfaces, where they induce membrane thinning and leakage [2].

Molecules blocking Aβ channel formation and other forms of membrane disruption have the potential to alleviate Aβ-induced cytotoxicity [4]. Current amyloid inhibitors target Aβ oligomers and fibrils outside the membrane. These amyloid inhibitors have been shown to be less effective at the membrane surface [4]. Therefore, it is very important that amyloid inhibitors have equal or superior efficacy at the peptide–membrane interface. Antibodies against Aβ in monomeric, oligomeric or fibrillar form have been developed [5,6]. However, these antibodies were raised for non-membrane amyloid peptides and therefore may neither decrease the rate of toxic Aβ aggregate formation in membranes nor inhibit amyloid membrane channel formation; thus, they may only decrease the number of existing aggregates outside the membrane.

The new concept, based on our studies of membrane RNAs [7,8,9,10,11,12], is to search for membrane RNA aptamers specific for membrane amyloid peptides. The RNA aptamers are selected for amyloid-42 (42 amino-acid long amyloid peptide) molecules, recruited to the membrane surface during the lipid bilayer formation. We also present the potential applications of these aptamers for exosome-based antioxidant strategies.

## 2. Results

### 2.1. Distribution of Aβ42 and RNA between Liposomes and Buffer Solution

We prepared several different pools of liposomes (as model membranes), made of various lipid compositions, in order to evaluate the distribution of amyloid peptide Aβ42 and RNA between liposomal membranes and buffer solution. Amyloid-liposomes were prepared from a mixture of amyloid peptide and lipids in organic solvents dried under a vacuum. The mole ration of Aβ42 to lipid was 2:100. This means that initially, each 100-nm liposome consisted of ca. 63,000 lipid molecules (assuming the mean surface area of lipid molecule ca. 1 nm^2^) and ca. 1250 Aβ42 molecules. After liposome preparation, a pool of the Aβ42 molecules dissociates from the liposomal membrane and equilibrium was reached in the distribution of Aβ42 between liposomes and the buffer. In order to observe the distribution of Aβ42 and RNA between liposomes and the buffer, liposomes were modified with a blue fluorescent dye (Pacific Blue-PE), incorporated into the liposomal membrane through the phospholipid PE (phosphatidylethanolamine). Amyloid peptide Aβ42 was modified with a fluorescein group attached to the N-terminus. In cells, the N-terminus is located outside the membrane during the cleavage of the amyloid precursor proteins by two proteolytic enzymes (β- and γ-secretases), while the C-terminus is at the end of hydrophobic residues located inside the membrane [3]. Therefore, the attachment of the hydrophilic fluorescent probe to the N-terminus of Aβ42 did not interfere with the interactions of the hydrophobic segment of Aβ42 with the hydrophobic part of the membrane. RNA 50N molecules (91-nt RNA molecules containing a central 50 bp region, random in sequence) were radio-labeled with ^32^P-GTP.

**Figure 1:**
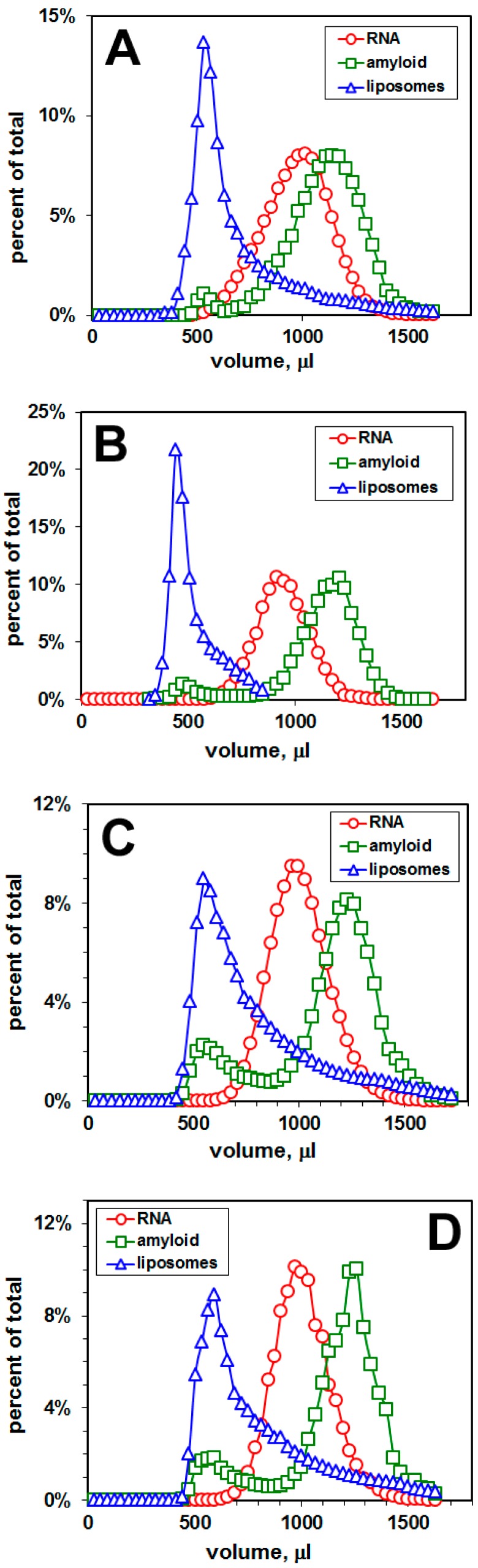

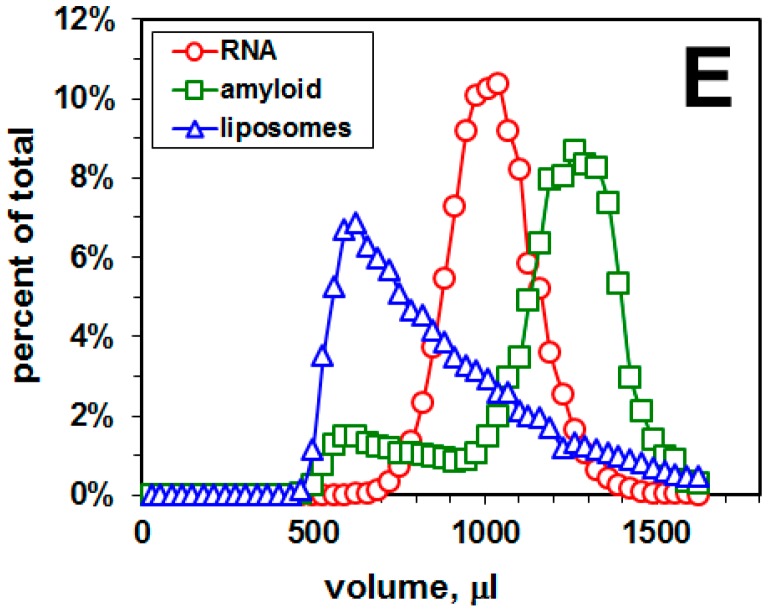
Distribution of amyloid peptide Aβ42 and a random pool of RNA sequences (RNA 50N) between liposomal membranes and buffer, measured by gel filtration. Elution profiles: (**A**) Rafted liposomes (lipids:Aβ = 100:2, mole ratio; lipids: DOPC:SM:CHOL = 6:3:1, mole ratio) modified with a blue dye Pacific Blue-PE; amyloid—Aβ42, modified with a fluorescein group attached to the N-terminus; (**B**) DOPC/DOPS liposomes (lipids:Aβ = 100:2, mole ratio; lipids: DOPC:DOPS = 10:1, mole ratio); (**C**) DOPC/DOPS liposomes (lipids:Aβ = 100:2, mole ratio; lipids: DOPC:DOPS = 10:2, mole ratio); (**D**) DOPC/DOPS liposomes (lipids:Aβ = 100:2, mole ratio; lipids: DOPC:DOPS = 10:3, mole ratio); (**E**) DOPC/DOPS liposomes (lipids:Aβ = 100:2, mole ratio; lipids: DOPC:DOPS = 10:4, mole ratio). Liposomes were modified with Pacific Blue-PE, while amyloid peptide was modified with a fluorescein group attached to the N-terminus. RNA 50N (91-nt RNA molecules containing a central 50 bp region, random in sequence) were radio-labeled with ^32^P-GTP. The RNA concentration was 0.5 μM and the liposome concentration was 10 mg/mL.

Figure 1 shows the elution profiles of the dispersion of amyloid-liposomes and RNA in the case of rafted liposomes and DOPC/DOPS liposomes, both containing Aβ42. The Sephacryl S-1000 column separates liposomes from RNA, and from amyloid peptide. As a result, three distinct elution profiles, shifted relative to each other, were obtained from each preparation: the elution of liposomes (measured as a fluorescence intensity of Pacific Blue), the elution of amyloid peptide (measured as a fluorescence intensity of fluorescein), and the elution of RNA (measured as a radioactivity of ^32^P-GTP within the RNA chain).

There is no binding of RNA 50N to the liposomal membranes, since no RNA peak associated with liposomes was observed (Figure 1). However, there are two peaks of Aβ42: One peak is associated with liposomes and another—representing amyloid peptide—not associated with liposomes. The percentage of the fluorescent amyloid peak associated with liposomes was 3.5% for rafted liposomes, and 6.1%, 18.2%, 16.5%, 16.6% for DOPS:DOPC-liposomes, mole ratio 1:10, 2:10, 3:10, 4:10, respectively. The increased percentage of negatively-charged DOPS within the liposomal membrane shifts the distribution of amyloid peptide Aβ42 towards the membrane. Given that Aβ42 peptide has an isoelectric point of 5.1, this effect cannot be solely due to an electrostatic phenomenon. The increased percentage of amyloid peptide in DOPS-containing liposomes affects the elution profile of liposomes (Figure 1).

### 2.2. Selection of a Pool of RNA Aptamers to Model Membranes Containing Aβ42

**Figure 2:**
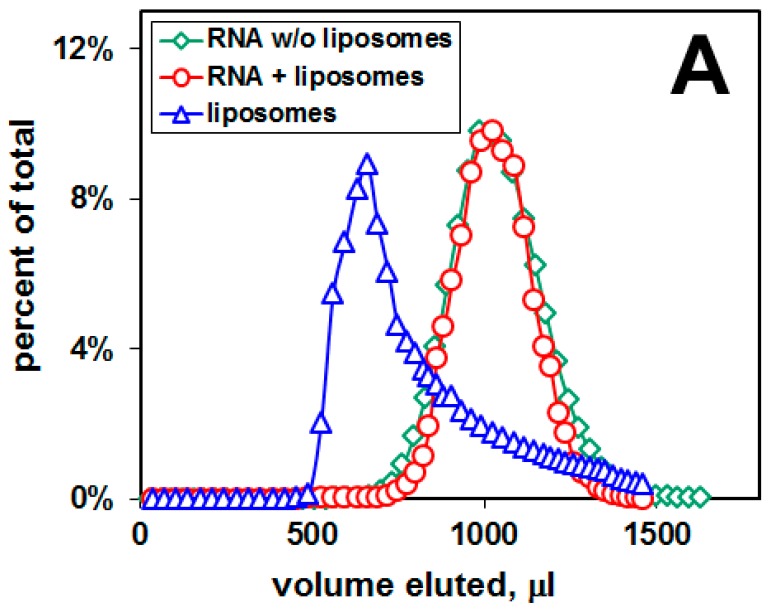

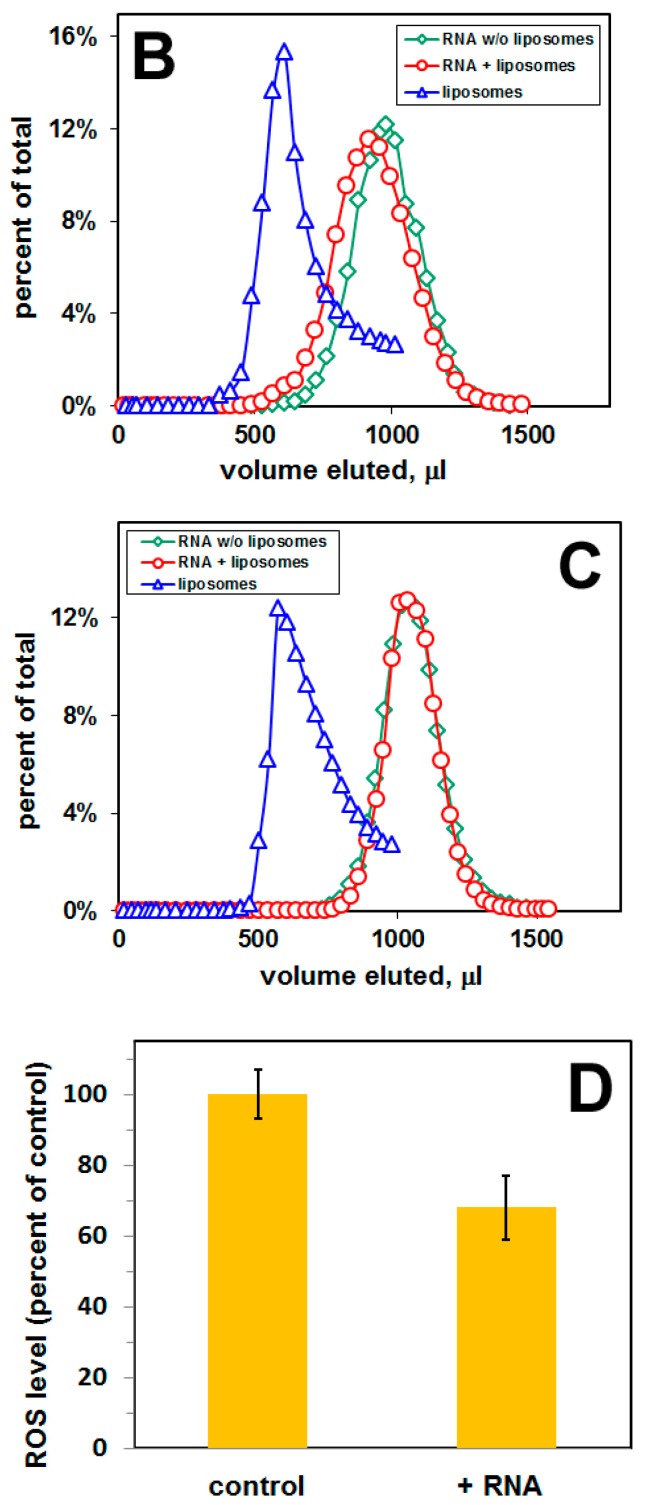
RNA binding to DOPC/DOPS liposomal membranes and changes in the level of reactive oxygen species (ROS). Elution profiles obtained by gel filtration: (**A**) Before cycle 1 (lipids:Aβ42 = 100:2, mole ratio; lipids: DOPC:DOPS = 10:3, mole ratio), membranes containing Aβ; (**B**) after cycle 8 (lipids:Aβ42 = 100:2, mole ratio; lipids: DOPC:DOPS = 10:3, mole ratio), membranes containing Aβ; (**C**) after cycle 8 (DOPC:DOPS = 10:3, mole ratio), membranes without Aβ. The eluted fractions were analyzed for ^32^P RNA content by scintillation counting, and for liposomes by A_320_ turbidity. RNA concentration 0.5 μM, liposome concentration 10 mg/mL. (**D**) The level of ROS in neuroblastoma cells after 24 h exposure to the pool of RNA aptamers.

We performed the selection-amplification procedure in order to select a pool of RNA aptamers specific to amyloid peptide embedded in lipid bilayers (Figure 2).

Before cycle 1, there was no binding of the pool of RNA 50N to the liposomal membranes containing Aβ42 (Figure 2A). After 8 cycles, the pool of selected RNA aptamers was tested for binding to Aβ42-containing liposomal membranes. Figure 2B shows that the “RNA+liposomes” peak is shifted toward the liposomal peak with a visible shoulder containing ca 2.7% of RNA radioactivity, indicating that these RNA molecules are bound to liposomal surface and they co-elute with liposomes. In contrast, there was no binding of this RNA pool to control liposomes, which have the same lipid composition but do not contain Aβ42 (Figure 2C), thus demonstrating binding of this pool of RNA aptamers to membrane amyloid peptide. 

Figure 2D shows that neuroblastoma cells treated with RNA- and Aβ-bound liposomes have the intracellular ROS level reduced to 68% ± 9% (mean ± SEM of three experiments) of the ROS level of cells exposed to Aβ-bound liposomes. Cells were treated with RNA- and Aβ-bound liposomes (taken from the liposomal peak of the elution profile, Figure 2B). As a control, cells were treated with Aβ-bound liposomes without RNA (taken from the liposomal peak of the elution profile without RNA). The in vitro cell-based experiments were based on the DCF assay.

## 3. Discussion

### 3.1. Membrane Affinity of Aβ Peptide

To characterize the Aβ42–membrane interaction, we measured the distribution of amyloid peptide (and also a random pool of RNA sequences, RNA 50N) between liposomal membranes and buffer. We quantified all the components using ^32^P-radioactivity measurements for RNA, and fluorescence intensities for both liposomes and Aβ42 (Figure 1). To better associate Aβ42 membrane affinity with specific lipid structures, we used liposomes made from pure lipids and Aβ42. Liposomes composed of an unsaturated lipid, sphingomyelin, and cholesterol exhibit phase separation in which sphingomyelin and cholesterol form liquid-ordered domains, often used as a model for membrane lipid rafts [10]. We also used liposomes, made from a mixture of synthetic phosphatidylcholine (PC) and phosphatidylserine (PS) at different mole ratios. Recent studies indicate that lipid rafts play an important role as pathological signaling platforms where receptors for Aβ oligomers are assembled [13]. It was also found that distinctive cell sensitivity to Aβ was essentially associated with cell membrane Aβ binding and this binding was correlated to the level of membrane-surfaced PS, thus indicating that PS may be one of the surface receptors that establish cell selectivity toward Aβ [14].

We employed gel filtration that separates voided liposomes from included Aβ42. In our study, we found that the affinity of Aβ42 is higher to PS-containing liposomes in comparison with rafted liposomes. In contrast, the random pool of RNA sequences (RNA 50N) had very low affinity both to rafted and PS-containing liposomes. The obtained data demonstrate that up to ca. 20% (depending on the percentage of anionic lipids in the membrane) of amyloid peptide, generated in the plasma (or intracellular) membrane by proteolytic cleavage of amyloid precursor proteins (APP) by β- and γ-secretases, can stay within the membrane, while over 80% of the peptide is released into the extracellular solution (or into cytoplasm). These results are consistent with the finding of Bokvist et al. [15] that acidic lipids can prevent the release of membrane-inserted Aβ, by stabilizing its hydrophobic transmembrane C-terminal part in an a-helical conformation via an electrostatic anchor between its basic Lys28 residue and the negatively charged membrane interface. Therefore, Aβ peptide can be firmly anchored in a membrane upon proteolytic cleavage.

Besides the membrane Aβ peptide generated by proteolytic cleavage, soluble Aβ oligomers in the brain specifically bind neuron plasma membrane, evoking neurotoxicity and synapse deterioration [13]. The propensity of Aβ to form weakly stable α-helical configurations that anchor the peptide to the membrane is regarded as important factor to favor membrane binding [16]. The membrane Aβ peptides are localized at the interface between membrane and water, with the C-terminal helix penetrating into the membrane core while the polar N-terminal region interacts mainly with the bilayer surface [16].

### 3.2. RNA Aptamers for Membrane Aβ42 Peptide

Above, we have measured that up to 18% of Aβ42 peptide can stay in the lipid bilayer after liposome formation. Assuming there are ca. 30,000 lipid molecules on the liposomal surface (per one 100-nm-liposome), there is initially 2 mol% = ca 600 Aβ42 molecules within one liposomal membrane, and ca. 300 Aβ42 in the external leaflet per liposome. We used DOPS:DOPC liposomes, mole ratio 3:10, in the selection-amplification procedure, and we found that ca. 16.5% of Aβ42 stay within the liposomal membrane. Therefore, we can estimate that during the selection, there are ca. 50 Aβ42 molecules within the outer layer of one liposome as a target for RNAs aptamers.

In order to show that a pool of RNA 50N binds membrane Aβ42, we used the selection-amplification (SELEX) method and gel filtration technique on a column that voids liposomes but includes RNAs of this size. RNA aptamers are single-stranded oligonucleotides usually composed of ca. 20 to 100 nucleotides [11]. Their unique three-dimensional structures confer specificity for binding to the target. A growing number of RNA aptamers have been selected experimentally using the SELEX approach, and these aptamers have several advantages over monoclonal protein antibodies or peptides with respect to their applications in medicine. Relatively few successful selections have been reported for membrane molecular targets, in contrast to the non-membrane molecular targets [11].

RNA (or DNA) aptamers were previously selected only against a non-membrane Aβ40 peptide (neither Aβ42 nor membrane Aβ). These aptamers were selected against monomeric/oligomeric forms of amyloid peptide, and they showed very low affinity to amyloid peptide in these forms; however, they exhibited a strong non-specific affinity to amyloid high-molecular weight fibrils [17,18]. Therefore, these aptamers can be used to detect/visualize amyloid fibrils, but not monomeric or low-molecular-weight oligomeric peptide. In addition, they can neither prevent formation of toxic amyloid oligomers from monomers in solution, nor inhibit formation of these aggregates within membranes. Aptamers selected to Aβ40 conjugated to gold nanoparticles are the only ones displaying a binding affinity to an oligomeric model of non-membrane Aβ40 with very low affinity [19]. However, the reported K_D_ values remain questionable, and they were not tested for their cross-reactivity with fibrillary assemblies of Aβ or of other amyloidogenic proteins [20].

Below we demonstrate the potential applications of membrane RNA aptamers to amyloid beta peptide for exosome-based antioxidant strategies, using exosomes. Exosomes (one of the form of extracellular vesicles, EV) are nanovesicles that originate from the endosome and are released from cells [21,22]. The size of exosomes (50–100 nm in diameter) is similar to the size of the liposomes we used for the selection of RNA aptamers for membrane amyloid peptide. Exosomes seem to have many features of an ideal carrier system for drug delivery to the brain: the exosome cargo is protected by the exosomal membrane from degradation in the circulation, exosomes seem to possess intrinsic cell targeting properties, they are able to overcome the blood–brain barrier (BBB), and they may be nearly non-immunogenic [23]. Moreover, exosomes utilize endogenous mechanisms for uptake, intracellular trafficking, and delivery of their content to recipient cells. Furthermore, clinical trials using EVs for immunotherapy have demonstrated the safety of EV administration in humans. Recent studies have also demonstrated that exosomes represent viable therapeutic targets against AD [24].

### 3.3. Membrane RNA—Exosome-Based Antioxidant Strategies: The Case of Membrane Amyloid Peptide Aβ42 at the Extracellular Leaflet of Plasma Membrane

In the diseased brain of AD patients, the level of soluble amyloids is significantly elevated. This could result in an accumulation of amyloid in the cerebrospinal fluid, insertion of amyloid peptide into membranes, and the formation of calcium-permeable amyloid channels in the cell plasma membrane. The membrane amyloid peptide can also be generated by proteolytic cleavage of APP by β- and γ-secretases. It is well accepted that neuronal death in AD is related to disturbances in Ca^2+^ homeostasis. The formation of Ca^2+^ channels in lipid bilayers was directly observed [16,25].

**Figure 3:**
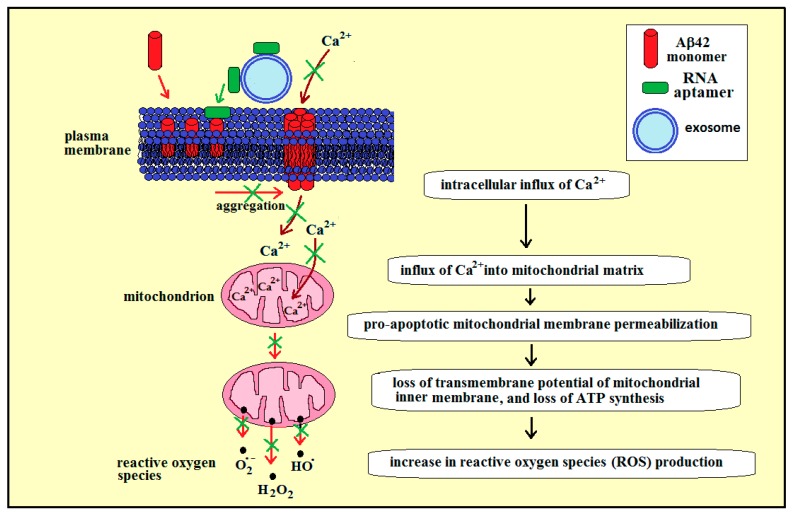
Proposed strategy for using RNA aptamers specific to membrane Aβ42 (42 amino-acid-long amyloid β-peptide) to reduce oxidative stress in Alzheimer’s disease, in the case of amyloid peptide Aβ42 at the extracellular leaflet of plasma membrane. Red arrows—Aβ42 aggregation and channel-formation, leading to an influx of calcium ions into the cytoplasm and into mitochondrial matrix (brown arrows), and production of reactive oxygen species (ROS) by mitochondria. RNA aptamers, initially bound to exosomes, can be transferred to the plasma membrane amyloid, which may prevent (green crosses) the channel formation, influx of calcium ions and ROS production.

Figure 3 presents our proposed strategy for using RNA aptamers specific to membrane Aβ42 to reduce oxidative stress in AD, in the case of amyloid peptide Aβ42 at the extracellular leaflet of plasma membrane. The increased concentration of calcium ions in cytoplasm can lead to an influx of these ions to mitochondria (Figure 3, brown arrows), which may cause the mitochondrial membrane permeabilization [26,27]. More specifically, the excess of intracellular calcium can be taken up by mitochondria through the mitochondrial calcium uniporter at the inner mitochondrial membrane [28], which can induce the opening of mitochondrial permeability transition pores (PTP, a multiprotein complex built up at the contact site between the inner and outer mitochondrial membranes), a phenomenon characterized by a sudden increase in the permeability of the inner mitochondrial membrane [29]. Following this, the caspase-dependent effects lead to a loss of transmembrane potential of the inner membrane and ATP synthesis, and an increase in reactive oxygen species (ROS) production (Figure 3, red arrows).

RNA aptamers specific for lipid rafts have been identified [10], and bifunctional RNA aptamers have been constructed from an RNA membrane aptamer and an RNA aptamer specific for tryptophan. These aptamers preserved their affinity to both membranes and the amino-acids [8]. Therefore, a bifunctional RNA aptamer can be constructed from two RNA aptamers: one specific for lipid rafts, and another specific for membrane amyloid peptide. Since the lipid bilayer of the exosomal membrane is enriched in raft lipids [30], the bifunctional aptamer containing two distinct regions (the first region with an affinity to lipid rafts and the second region with an affinity to the amyloid peptide) can be transported to the brain while attached to the lipid rafts of the external surface of the exosomes, cross the BBB while attached to the exosome, and can then be transferred to a membrane amyloid peptide at the extracellular leaflet of neural plasma membrane due to its affinity with the membrane amyloid peptide (Figure 3, green arrow). Following this, the RNA aptamer bound to membrane amyloid peptide can inhibit formation of toxic amyloid aggregates and amyloid calcium channels within the plasma membrane, thus reducing the calcium-dependent generation of ROS by mitochondria in the neural cells in the brain of AD patients (Figure 3, green crosses).

### 3.4. Membrane RNA—Exosome-Based Antioxidant Strategies: The Case of Membrane Amyloid Peptide Aβ42 at the Cytoplasmic Leaflet of Intracellular Membranes

**Figure 4:**
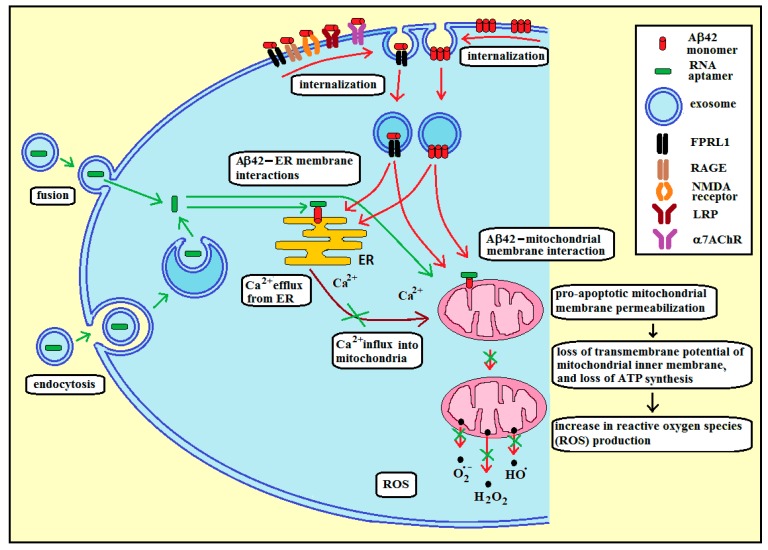
Proposed strategy for using functional exosomes with RNA aptamers specific to membrane Aβ42 (42 amino-acid-long amyloid β-peptide) to reduce oxidative stress in Alzheimer’s disease, in the case of intracellular amyloid peptide Aβ42. Amyloid peptide can enter cytoplasm through endocytosis (red arrows), or can be produced at the intracellular membranes by β- and γ-secretases. The interaction of amyloid peptide with ER (endoplasmic reticulum) membrane (red arrows) can lead to the release of calcium ions by ER and mitochondrial uptake of these ions (brown arrows), resulting in mitochondrial membrane permeabilization, loss of transmembrane potential of the inner mitochondrial membrane, and mitochondrial ROS production. Similar effects can arise from the direct interaction of amyloid peptide with mitochondrial membranes (red arrows). RNA aptamers can enter cytoplasm through the fusion of RNA-containing exosomes with the plasma membrane, or through endocytosis of these exosomes. RNA aptamers can bind to membrane amyloid, thus preventing (green crosses) ER/mitochondria membrane permeabilization, efflux of calcium ions from ER, and ROS production by mitochondria.

Figure 4 presents our proposed strategy for using RNA aptamers specific to membrane Aβ42 to reduce oxidative stress in AD, in the case of amyloid peptide Aβ42 at the intracellular leaflet of plasma membrane.

In order to deliver RNA aptamers into the cytoplasm, the RNA molecules have to be encapsulated inside exosomes. Through endocytosis of exosomes by neural cells and the subsequent fusion of exosomal membrane with the endosomal membrane, RNA aptamers can be released into the cytoplasm and target membrane amyloid peptides located at the cytoplasmic leaflet of intracellular membranes (Figure 4, green arrows). Loading of exosomes with RNA aptamers can be achieved through incubation at room temperature, repeated freeze-thaw cycles, extrusion, sonication, electroporation, or treatment with a detergent-like molecule (e.g., saponin) [23]. Electroporation, which is based on spontaneous pore formation after stimulation with an electrical signal, was used previously to equip dendritic cell-derived exosomes with short interfering RNA (siRNA) for delivery to the brain, including crossing the blood–brain barrier (BBB) [31]. Exosome delivery of siRNA resulted in a significant and dose-dependent knockdown of mRNA for BACE1, a protease that produces N-terminal cleavage of amyloid precursor proteins that lead to Aβ aggregations. Thus, targeted exosomal delivery of siRNA has the potential to cross the blood–brain barrier to reach the brain and generate specific knockdown to alleviate the pathogenesis of AD. [24]. Interestingly, cells able to produce neuron-targeted exosomes over a prolonged time frame were generated. These cells were genetically engineered to boost the efficiency of exosome delivery. These exosomes could cross the BBB and consistently deliver cargo mRNA to the brain [32].

The intracellular amyloid peptide (iAβ) can be generated inside neural cells, or can appear in the cytoplasm as a result of a reuptake of extracellular Aβ (eAβ) (Figure 4, red arrows). Aβ42 is most often located in the perinuclear region, and it is found in the outer membranes of multivesicular bodies (MVBs), in endoplasmic reticulum (ER), endosomes/lysosomes, and mitochondria of neuronal cells, where it is implicated in synaptic pathology [33,34]. Expression of iAβ precedes extracellular plaque deposition. In addition, the cognitive deficits, behavioral changes, and axonal degeneration associated with AD-type pathologies, are more closely linked to intraneuronal accumulation of Aβ oligomers than that of extracellular plaque deposition [35,36]. Therefore, this accumulation may be an early event in the progression of AD pathologies. At the cellular and molecular level, iAβ accumulation has been shown to impair aspects of axonal transport and synaptic transmission, thereby suggesting a role in the cognitive impairment associated with AD. APP cleavage by β-and γ-secretases (resulting in the production of Aβ42) is thought to occur in the endoplasmic reticulum (ER) and trans-Golgi network (TGN) and secreted as part of the constitutive secretory pathway [37]. Aβ is also produced in the endosomal/lysosomal system and within the mitochondria [38]. Both membrane- and soluble-Aβ can be shuttled between cellular compartments using transport vesicles.

Extracellular amyloid can be internalized through two different pathways: Endocytosis of an Aβ membrane-receptor complex or endocytosis of membrane Aβ aggregates (Figure 4, red arrows). The following receptors were found to be involved in the Aβ42 internalization: the α7 nicotinic acetylcholine receptor (α7nAChR), the low-density lipoprotein receptor-related protein (LRP), N-methyl-d-aspartate (NMDA) receptors, the scavenger receptor for advanced glycation end products (RAGE), and the formyl peptide receptor-like 1 (FPRL1, a G-protein-coupled receptor) [37]. It was demonstrated that membrane aggregates of Aβ were taken up and accumulated in endocytic vesicles. [39]. In addition, Aβ42 could be internalized into lysosomes/endosomes, mitochondria and endoplasmic reticulum in the cortex and CA1 region of the hippocampus. [40].

The amyloid-induced stress in the endoplasmic reticulum results in the release of calcium ions into the cytoplasm, which are taken up by mitochondria (Figure 4, brown arrows) [28,34,41]. There is also an additional direct effect of iAβ resulting from the interaction of iAβ (delivered from other compartments or produced inside mitochondria) with mitochondrial membranes. These interactions can lead to (a), an increase in the opening probability of the mitochondrial PTP and subsequent decrease in mitochondrial membrane potential [42]; (b), an increase in the outer membrane permeability through amyloid channel formation, and subsequent ionic imbalance resulting in mitochondrial swelling and activation of proteolytic enzymes (calpain and caspase) [43]; and (c), the direct gradual damage of mitochondrial membranes, ultimately leading to apoptosis [34]. Thus, two independent iAβ-related factors can cause the final mitochondrial membrane permeabilization, loss of transmembrane potential of the inner mitochondrial membrane, mitochondrial ROS production, and apoptosis: The first factor being mitochondrial uptake of calcium ions, released by ER, due to the interaction of amyloid peptide with ER membrane; and the second factor being a direct interaction of amyloid peptide with mitochondrial membranes.

In Down’s syndrome, adults have a high level of Aβ peptide deposition in brain [44]. This is attributable to triplication and overexpression of the gene for Aβ precursor protein, located on chromosome 21 [45]. Therefore, these adults have a high risk of developing AD in a progressive age-dependent manner and as such are at high risk for the development of dementia [44]. In addition, there is ample evidence of the involvement of oxidative stress in the pathogenesis of both Down’s syndrome and AD [46]. Therefore, RNA aptamers specific for membrane Aβ could potentially serve both in AD treatment and as dementia-preventive therapy for children and young adults with Down’s syndrome.

## 4. Materials and Methods

### 4.1. Materials

Chemicals were analytical-reagent grade whenever available. Sephacryl S-1000 was obtained from Pharmacia. The following were purchased from Avanti Polar Lipids (Alabaster, AL, USA): 1,2-dioleoyl-sn-glycero-3-phosphocholine (DOPC); 1,2-dioleoyl-sn-glycero-3-phospho-l-serine (DOPS), cholesterol (CHOL); *N*-stearoyl-d-erythro-sphingosylphosphorylcholine (Stearoyl Sphingomyelin, SM). H_2_DCF-DA (2′,7′-dichlorodihydrofluorescein) and fluorescent membrane probe Pacific Blue-PE were obtained from Invitrogen Molecular Probes (Eugene, OR, USA). The amyloid peptides Aβ42 and Aβ42, modified with a fluorescein group attached to the N-terminus, were purchased from Biopeptide (San Diego, CA, USA). T7 RNA polymerase (used for in vitro transcription) was obtained from Epicentre (Madison, WI, USA).

### 4.2. Preparation of Large Unilamellar Vesicles (LUV)

The appropriate lipids were dissolved in chloroform or chloroform/methanol (2/1), mixed with amyloid peptide solution in DMSO. Following this, solvents were evaporated under a stream of nitrogen gas, the pellet was desiccated under a vacuum for at least 2 h and resuspended in AM buffer (25 mM HEPES, pH 7.33, 118 mM NaCl, 4 mM MgCl_2_, and 2 mM CaCl_2_, 3 mM KCl, 294 mOs/L), and multilamellar liposomes containing Aβ42 were formed by gentle vortex. The suspension was subjected to seven freeze–thaw cycles by repeated immersion in liquid nitrogen followed by warming in 60 °C water. LUV containing Aβ42 were prepared by extrusion using the Avanti MiniExtruder (Alabaster, AL, USA) with a filter pore diameter of 100 nm. The composition/pH/osmolarity of AM buffer was based on the ion composition/pH/osmolarity of the cerebrospinal fluid. In a procedure reported earlier [47], lipid in organic solvent was added to a thin film of peptide at the beginning of liposome preparation

### 4.3. Distribution of Aβ42 between Liposomes and Buffer

LUV containing fluorescent probe Pacific Blue-PE and amyloid peptide Aβ42, modified with a fluorescein group attached to the N-terminus, were prepared. Incubation of internally ^32^P-labeled RNA 50N (1.0 nmol, random pool of RNA sequences) with LUV (20 μL, 10 mg/mL) was performed in AM buffer at room temperature for 5 min, followed by gel filtration on a Sephacryl S-1000 1-mL column. The eluted fractions were analyzed for ^32^P RNA content by scintillation counting, and for liposomes and amyloid by Pacific Blue fluorescence intensity and fluorescein fluorescence intensity, respectively, using NanoDrop spectrofluorometer (Madison, WI, USA).

### 4.4. Selection Procedure

The selection was performed according to Vlassov et al. [48]. Briefly, incubation of internally ^32^P-labeled RNA (1.0 nmol in the first selection round, decreasing in subsequent rounds) with liposomes (20 μL, 10 mg/mL) was performed in AM buffer at room temperature for 5 min, followed by gel filtration on a Sephacryl S-1000 1-mL column. The initial RNA pool with 50-mer random region (50N) was generated by T7 polymerase by in vitro transcription of a DNA template strand of the sequence: 5′-TGG TCA TGT GAT CGG CGT ATG—50N—TAT CGT GTC ATC GTC GTC CCT ATA GTG AGT CGT ATT A-3′ (the underline sequence is the T7-promoter region). Approximately 10^15^ molecules of 91-nt RNA transcribed from independently synthesized DNA templates were heated in water at 65 °C for 5 min, 10xAM buffer was added, and solution was cooled to room temperature over 10 min to allow for RNA folding. The transcribed RNA 50N had the following sequence: 5′-TGG TCA TGT GAT CGG CGT ATG—50N—TAT CGT GTC ATC GTC GTC CC-5′.

**Figure 5:**
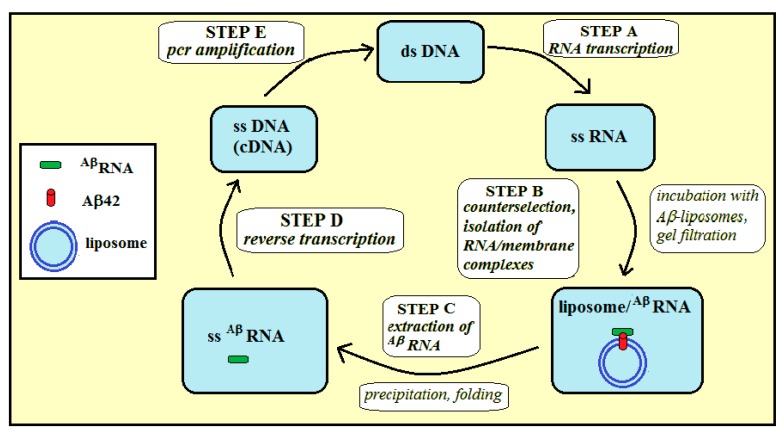
Steps of the selection-amplification (SELEX) method applied for RNA aptamers specific for membrane Aβ42 (42 amino-acid-long amyloid β-peptide).

The selection-amplification (SELEX) procedure (Figure 5) starts with step A: T7 RNA polymerase is used to transcribe dsDNA library into a single-stranded RNA library containing ca. 10^15^ sequences. In step B, ssRNA is first incubated (counterselection) alternatively with liposomes without Aβ42 (to remove RNA sequences that bind non-specifically to liposomes) or with buffer only (to remove non-specific RNA aggregates), then ssRNA is incubated with amyloid-liposomes, and next the amyloid/liposome/RNA complexes are separated from unbound species by gel chromatography using Sephacryl S-1000. The eluted fractions were analyzed for ^32^P RNA content by scintillation counting and for liposomes by A_320_ turbidity [10]. Co-elution of RNA and liposomes indicated binding. ^Aβ^RNA are RNA sequences that can bind to membrane amyloid peptides. In step C, ^Aβ^RNA sequences are extracted from the liposome suspension by precipitation. In step D, the ss^Aβ^RNA sequences are reverse transcribed to give a cDNA copy of the winning sequences (i.e., enriched in the RNAs that can bind membrane amyloid peptide). For step E, PCR amplification completes the selection cycle and provides a dsDNA template enriched in the winning sequences for the next cycle.

In order to improve the stability of RNA aptamers, both in cytoplasm and other fluids containing nucleases, it is possible in future applications to synthesize the aptamers using 2′-*O*-Me pyrimidines which are nuclease resistant [49] and inexpensive.

### 4.5. Cell Culture and Measurement of Reactive Oxygen Species (ROS)

The IMR-32 neuroblastoma cell line was purchased from the European Collection of Authenticated Cell Cultures (ECACC), a Culture Collection of Public Health England (Salisbury, UK) through Sigma-Aldrich (Poznan, Poland), and were cultured in Eagle’s Minimum Essential Medium (Sigma) supplemented with 1% Non-Essential Amino Acids (NEAA), 2 mM glutamine, 0.5 mg/mL of streptomycin sulfate, 100 units/mL of penicillin G, and 10% fetal bovine serum (FBS) in a 5% CO_2_ and 95% air humidified atmosphere at 37 °C [50]. Changes in the level of reactive oxygen species (ROS) were measured using a fluorescent probe 2′,7′-dichlorodihydrofluorescin diacetate (H_2_DCF-DA) [51]. The detection is based upon the formation of a fluorescent dichlorofluorescein (DCF) from nonfluorescent H_2_DCF-DA. H_2_DCF-DA is incubated with cells and it enters the cells, then is cleaved by cytosolic esterases to 2′,7′-dichlorodihydrofluorescein, H_2_DCF (this prevents the back-diffusion of the dye to the extracellular space), and next is oxidized to the fluorescent DCF. Observed fluorescence is proportional to the concentration of intracellular ROS. Cells were cultured in 96-well plates, treated with RNA- and Aβ-bound liposomes (taken from the liposomal peak of the elution profile, Figure 2B) for 24 h in a 5% CO_2_ and 95% air humidified atmosphere at 37 °C. As a control, cells were treated with Aβ-bound liposomes without RNA. After the incubation period, cells were washed with PBS and stained with 2.5 μM H_2_DCF-DA for 15 min. After staining, the dye solution was removed and the cells were washed with PBS. Fluorescence of DCF was quantified using Cary Eclipse spectrofluorometer with a microplate reader with excitation and emission wavelength of 485 and 530 nm, respectively. Untreated cells were used for background readings.

## 5. Conclusions

In this study, we searched for RNA aptamers specific for membrane amyloid peptides. The results demonstrate that up to ca. 20% of amyloid peptide, generated in the plasma (or intracellular) membranes as a result of proteolytic cleavage of amyloid precursor proteins by β- and γ-secretases, can stay within the membrane. Functional exosomes containing the selected pool of aptamers can inhibit the formation of toxic Aβ aggregates at the site of their nucleation, or can inhibit amyloid channel activity, and reduce reactive oxygen species production by mitochondria. RNA aptamers specific for membrane Aβ could potentially serve both in AD treatment and as dementia-preventive therapy for children and young adults with Down’s syndrome.

## Abbreviations

AD	Alzheimer disease
Aβ	amyloid β-peptide
Aβ42	42 amino-acid-long amyloid β-peptide
APP	amyloid precursor protein
BBB	blood-brain barrier
CHOL	cholesterol
ΔΨm	transmembrane potential
DCF	dichlorofluorescein
DMSO	dimethyl sulfoxide
DOPC	1,2-dioleoyl-sn-glycero-3-phosphocholine
eAβ	extracellular amyloid peptide
ER	endoplasmic reticulum
iAβ	intracellular amyloid peptide
LUV	large unilamellar vesicles
MVBs	multivesicular bodies
PC	phosphatidylcholine
PS	phosphatidylserine
PTP	permeability transition pore
ROS	reactive oxygen species
SELEX	systematic evolution of ligands by exponential enrichment
siRNA	short interfering RNA
SM	*N*-stearoyl-d-erythro-sphingosylphosphorylcholine (stearoyl sphingomyelin)
TGN	trans-Golgi network
